# Cost of Provisions in Hospitals

**Published:** 1906-01-13

**Authors:** 


### COST OF PROVISIONS IN HOSPITALS.

*To the Editor of* The Hospital.

Sir,—I am desirous of having an expression of opinion from you or from some of your numerous readers as to a proper expenditure per head per day or week for food of all descriptions—including beef-tea as required—for a provincial hospital with seventy beds and a staff of all grades numbering thirty-five—say two resident surgeons, matron, and deputy, twenty nurses, 11 domestics.

Yours, etc.,

Weekly Board.

*** A series of articles dealing at considerable length with the Cost of Provisions in Institutions appeared in The Hospital in 1904. These would probably supply our correspondent with the information he requires.—Ed. The Hospital.

